# Exploration of Evaluation Practices in Social Prescribing Services in Ireland: A Cross-Sectional Observational Study

**DOI:** 10.3390/healthcare12020219

**Published:** 2024-01-16

**Authors:** Hayley Connolly, Natalie Delimata, Karen Galway, Bridget Kiely, Margaret Lawler, Jill Mulholland, Megan O’Grady, Deirdre Connolly

**Affiliations:** 1 Discipline of Occupational Therapy, School of Medicine, Trinity College Dublin, F91 YW50 Sligo, Ireland; connolha@tcd.ie (H.C.); lawlerm1@tcd.ie (M.L.); 2 Department of Social Sciences, Atlantic Technological University, F91 YW50 Sligo, Ireland; delimata.natalie@itsligo.ie; 3 School of Nursing and Midwifery, Queen’s University Belfast, Belfast BT9 7BL, UK; k.galway@qub.ac.uk (K.G.); jmulholland18@qub.ac.uk (J.M.); 4 Department of General Practice, RCSI University of Medicine and Health Sciences, D02 YN77 Dublin, Ireland; bridgetkiely@rcsi.ie; 5 Discipline of Physiotherapy, Trinity Centre for Health Sciences, St. James’ Hospital, D08 W9RT Dublin, Ireland; ogradyme@tcd.ie

**Keywords:** social prescribing, evaluation methods, approaches, measurement, barriers and facilitators, core outcome set

## Abstract

National health services in Ireland and the UK fund the majority of social prescribing services and have issued recommendations for evaluation. However, it is not known what outcomes are prioritised for evaluation within individual services and what evaluation methods are used to capture recommended outcomes. A survey was carried out to examine evaluation practices of social prescribing services on the island of Ireland. This study used a cross-sectional observational design. The sample was all the staff involved in delivering and/or managing SP services on the island of Ireland. Questionnaires were distributed at a national SP conference and online. Closed-response questions were analysed using descriptive statistics. Content analysis was used for open-ended questions. Eighty-four usable surveys were returned (50% from the Republic of Ireland and 50% from Northern Ireland). All respondents (100%) agreed on the importance of measuring SP outcomes. The most frequently measured outcomes were health and well-being (89.2%) and loneliness (84%). The least frequently measured outcome was the satisfaction of healthcare professionals referring to SP: 78.4% of respondents never measured this outcome. The most frequently used measurement tool was the Short Warwick Edinburgh Mental Well-Being Scale, with 38/76 (50%) respondents using this measure. There was a lack of standardised measures identified for some outcomes. For example, 70% of respondents reported always measuring physical activity (PA), but only four respondents identified a specific PA measure. In open-ended questions, respondents recommended flexibility in evaluation methods to reflect the complexity and individualised focus of SP. They also identified the need for protected time to complete evaluations and recommended a national strategy to inform priorities in evaluations. This study demonstrates a wide variation on the island of Ireland on how SP services are measuring outcomes, with many outcomes rarely or never measured using standardised measures. Agreement is needed on a core outcome set for social prescribing in order to guide service delivery and evaluations.

## 1. Introduction

Social prescribing is a non-medical, community-based service that supports people by linking them to non-clinical activities and services in their communities [1]. It is often provided for individuals living in disadvantaged communities, those with long-term chronic illnesses who require support to improve their physical and mental health, and individuals who are socially isolated [2]. Social prescribing services are designed differently according to the needs of the individuals for whom the services have been established. However, the process usually involves an individual being referred to a link worker in a social prescribing service, typically by a healthcare professional. The link worker then meets with the individual to determine what difficulties the individual is experiencing and what activities/services can best resolve any difficulties identified. Following this meeting, the link worker explores activities and/or services available in the individual’s local community to address the identified issues. Once the link worker identifies relevant services and/or activities, they then provide the contact information for the service or activity to the individual or, if necessary, will accompany the person to their initial attendance at the service or activity [2]. The amount and type of support a link worker provides varies due to the wide-ranging and complex issues they are required to address with each individual. Community services, to which individuals are linked, can range from exercise groups, counselling services, financial advice centres, education workshops, and art classes, depending on the individual’s preferences and on local activity options that are available in the community [3].

Social prescribing is growing in popularity and is driven by policies that seek to address the challenges that health systems face in delivering care to an ageing population, rising numbers of long-term conditions, a mental health crisis, and the impact of social determinants on health [4]. Social prescribing services are now widely established in Ireland, the UK, the Netherlands, Canada, Portugal, and China, amongst other countries [5]. In Ireland, the number of services has increased considerably over the past five years. There are currently approximately eighty-six services across the island of Ireland situated in a range of locations, including community health programmes, family resource centres, universities, hospitals, and prison services [6].

This ever-growing demand for social prescribing requires a robust evaluation to determine the impact of social prescribing on individuals’ health, cost-effectiveness, and the impact on local communities where services are based. A recent systematic review concluded that the economic evaluation of social prescribing is weak and that there is a lack of research and evidence in evaluating the impact of social prescribing [7]. No consistent evidence is currently published that supports how social prescribing interventions help improve individuals’ mental health, quality of life, physical function, and/or level of social support.

The absence of robust evidence is related to the complexity of social prescribing and the capacity of often small local organisations without an academic affiliation to conduct robust evaluations [8]. Holistic social prescribing is a complex intervention in that it is tailored to the individual and the context, acts on an individual and community level, and can produce a wide range of outcomes, therefore presenting challenges for comprehensive and inclusive evaluations [9]. Determining a common framework for evaluating social prescribing is problematic due to the variety of where and how services are located and delivered in different countries. There is no globally agreed framework for evaluating social prescribing services, which makes demonstrating their impact and building a body of international evidence challenging.

In England, the National Health Service’s (NHS) draft outcome framework recommends evaluating the impact of social prescribing on three levels: (i) individuals attending social prescribing, (ii) the community in which the service is located, and (iii) on local healthcare services [10]. However, it does not identify specific measures for evaluation at these three levels. Therefore, it is not known what approaches social prescribing staff use to evaluate their services and what specific measures they use. This reduces opportunities to synthesise research findings and to make recommendations on a national framework to guide optimal evaluation methods. In Ireland, the Health Service Executive (HSE) conducted an evaluability assessment with stakeholders and developed an outcomes framework that recommends that social connectedness and personal well-being should be evaluated as a minimum requirement [8]. However, since the publication of this framework, there has been no study to date of whether social prescribing services are implementing these recommendations when carrying out evaluations.

Many factors impact on how health services are evaluated. These include the knowledge and skills of staff who complete evaluations and the amount of support staff need, or have access to, in order to guide evaluation priorities and practices. There is, therefore, consensus that given the complex nature of social prescribing, different evaluation methods are required [8]. However, some of the variations in outcomes and measures used may not be necessary and may add to the challenge of establishing the impact of social prescribing services across Ireland. As a first step, more information is needed on the evaluation practices of social prescribing services. Therefore, the purpose of this study was to (i) identify what outcomes are being measured by social prescribing services, (ii) describe approaches being used by social prescribing services to evaluate outcomes, and (iii) to identify current barriers and facilitators to evaluation.

## 2. Methods and Materials

This study used a cross-sectional observational design. Cross-sectional observational studies are used to measure variables of interest at a particular point in time [11]. A commonly used method for collecting data in cross-sectional studies is through surveys, which was utilised for this research [11,12]. Ethical approval for this study was obtained from University of Dublin, Trinity College, School of Medicine Research Ethics Committee (approval no.: 20220406).

### 2.1. Participants

At the time of the survey, eighty-six individual social prescribing services were identified in the directory of social prescribing services on the All-Ireland Social Prescribing Network website [6]. Funders of social prescribing services on the island of Ireland include National Health Service (NHS), Health and Safety Executive (HSE), charitable organisations, community-based organisations, and other health-related statutory and voluntary organisations. Therefore, there is considerable variation in who attends social prescribing services and in service delivery models. Given this variation, it was important to include all social prescribing services in the study sample in order to capture variation in evaluation methods and practices across different services. Therefore, the sample for this study comprised all services involved in delivering social prescribing on the island of Ireland. There was no pre-determined sample size for this study, as the aim was to include all services across the island of Ireland.

### 2.2. Data Collection Methods

A survey was designed by the research team to meet the objectives of the study. The survey consisted of three sections: Part 1 explored the profile of the responding service. This included location, sources of referrals, reasons for referral, age group of service users and profile of community activities social prescribing services typically link services users. Part 2 asked respondents to identify outcomes measured by their service from a list of social prescribing outcomes, how frequently these outcomes are measured and what methods they use to measure these outcomes. Outcomes included in the survey are the recommended outcomes as outlined in the HSE Evaluability Framework [8]. The HSE Framework categorises SP outcomes into person-centred outcomes, social prescribing service-related outcomes and health utilisation outcomes. Part 3 of the survey examined barriers and facilitators to evaluation in social prescribing services and included an open-ended question inviting respondents to add any further information/feedback on their experiences of evaluating their social prescribing services and/or recommendations for evaluation. The survey was piloted for readability, relevance of questions and time to complete.

This study sought representation from all social prescribing services on the island of Ireland. Therefore, the survey was distributed via two methods. The first was through distribution of the survey at the annual conference of the All-Ireland Social Prescribing Network (AISPN) in 2022. As this was a national conference, it was expected that individuals attending the conference would include those responsible for managing and/or delivering social prescribing services across the island of Ireland and, therefore, would be informed about evaluation practices in their services. Attendees were invited to complete the survey if they were directly involved in managing and/or providing social prescribing services on the island of Ireland. On registration at the conference, all attendees were provided with a participant information leaflet and a hard copy of the survey. As only one survey was required per service, attendees were requested to liaise with colleagues from their service who might also be attending the conference to ensure that only one survey was submitted per service. Regular reminders were provided during the two-day conference requesting attendees to complete the survey. Respondents returned the survey to a secure postal box located at the conference registration desk.

However, as the AISPN conference was held in Northern Ireland, the majority of surveys collected during the conference related to services based in Northern Ireland. Therefore, in order to increase representation from services based in the Republic of Ireland, soft copies of the survey were distributed via Qualtrics after the conference. The All-Ireland Social Prescribing Network (AISPN) website contains a directory of social prescribing services in the island of Ireland [6]. Contact details of the listed services were taken from the website, and a link to the survey was emailed to all listed services. The survey link was also emailed to the Chair of the Irish Social Prescribing Link Worker Peer Network, who forwarded it to all members of the Network. A note was included in the online survey requesting services not to complete the survey if they had already completed a hard copy version of the survey at the AISPN conference and to request that only one person involved in delivering and/or managing social prescribing from each service should complete the survey.

### 2.3. Data Analysis

Quantitative data were analysed using SPSS 25 statistical software package. Descriptive statistics were used to report service characteristics, demographics of responding services and frequencies in approaches to evaluation methods. Both content analysis and frequency counts were used to analyse open-ended questions, e.g., ‘please provide the name of specific measurement tools used in your service’. Content analysis was used to analyse answers to open-ended questions in Part 3 of the survey [13]. This involved identifying response categories based on the aims of the study. All data were then coded against the agreed categories and were grouped into sub-categories. Analysis of open-ended questions was completed independently by two team members (DC and HC), who then met to compare codes and make decisions where disagreements arose.

## 3. Results

In addition to presenting quantitative results from the survey, findings from open-ended questions are integrated with quantitative findings.

### 3.1. Profile of Social Prescribing Services

A total of eighty-six surveys were completed, with eighty-four included in the analysis. Two surveys were not completed beyond Part 1 of the survey (i.e., service profile) and, therefore, were excluded from the study. The majority (69%, *n* = 58) were completed by individuals attending the conference, and the remaining were completed online. There was an equal number of respondents working in the Republic of Ireland (ROI) (*n* = 42) and in Northern Ireland (NI) (*n* = 42) (Table 1). Survey respondents identified their roles as social prescribing link workers (47.6%, *n* = 40), SP Coordinators (26.2%, *n* = 22) and social prescribing managers (26.2%, *n* = 22). The location of services was predominantly in community-based organisations (Table 1).

The majority of respondents reported receiving referrals from community mental health services (*n* = 69, 82.1%). The top two categories of healthcare professional referrals included General Practitioner [GP] practices (*n* = 67, 79.8%) and Social Workers (*n* = 59, 69.4%). Occupational therapists (*n* = 45, 53.5%) and physiotherapists (*n* = 42, 50%) also refer frequently to respondents’ services. Fifty-seven respondents (67.1%) identified receiving self-referrals. Almost all respondents reported that the majority of their service users are in the age category of 31–65 and 66+ years, with few people under 18 years attending their services (Table 1). Respondents were asked to identify the top five reasons for referral to their services. A wide range of reasons were identified, which were categorised into mental health, social health, physical health, and other (Table 1).

On examining reasons for referral within the categories of mental health, social health, physical health, and other, the most frequently reported reasons were within the categories of mental and social health (Table 2). Loneliness was identified as the most common reason for referral, with eighty respondents (95.2%) identifying this as the main reason for referral to their service. This was followed by mental health-related reasons (unspecified) (39%, *n* = 32), anxiety (34.5%, *n* = 29), and depression (34%, *n* = 28). The most common physical health-related reasons for referral were chronic disease management (19.5%, *n* = 19) and exercise (17%, *n* = 14). The least commonly reported reasons for referral included falls prevention (physical health) and financial support (Other).

### 3.2. Evaluation Methods

Regarding how often services collect evaluation data, 76% of respondents (*n* = 57/75) reported collecting baseline evaluation data at the first meeting with new service users. Follow-up data were collected between one and three months by forty-eight respondents (64%), and 38 respondents (50.7%) reported collecting follow-up data beyond three months.

For the purposes of this survey, social prescribing outcomes were categorised into service-user outcomes, service-related outcomes and healthcare-utilisation outcomes as per the Health Service Executive (HSE) Evaluability Framework [8]. For each of these outcomes, survey respondents were asked to identify (i) how frequently they evaluated each outcome, (ii) what approach/es they used for evaluation, and (iii) the name of standardised questionnaires used for measuring these outcomes. There was significant variation in the number of respondents answering each of these three questions (Table 3).

The most frequently measured service-user outcome was general health and well-being (GHWB), with 66/74 respondents (89.2%) reporting they always measure GHWB Sixty respondents reported that the most frequently used approaches to measuring GHWB were questionnaires and informal discussion. Forty-two of the seventy-four respondents identified eight different questionnaires for measuring GHWB, with the Short Warwick–Edinburgh Mental Well-being Scale (SWEMWBS) [14] the most frequently identified. The least frequently measured person-centred outcomes were employment and financial situation (Table 3).

The most frequently identified standardised measure used by respondents for measuring service-user outcomes was the SWEMWEBS [14], followed by the Outcome Star [15] (Table 4). Forty-nine respondents (57%) reported they use a digital platform to record evaluation data.

For service-related outcomes, 80.8% (*n* = 59) of respondents stated they record the number of referrals they receive for their service and 74% (*n* = 54) record the number of people referred to their service who do not attend. Satisfaction of health care professionals and community-based organisations who refer individuals to their services was the least frequently measured outcome (*n* = 15/74, 20.3%) (Table 5). The most frequently identified approaches for measuring service-related outcomes were questionnaires and informal discussion.

Outcomes related to healthcare utilisation included the number of GP and Emergency Department [ED] visits as reported by service users. Just over half of respondents stated they always evaluate the number of GP (*n* = 39/74, 52.7%) and ED visits (*n* = 38/75, 50.7%). Questionnaires were the primary approach used to measure both of these outcomes: *n* = 17/38 (44.7%) for GP visits and *n* = 15/36 (41.7%) for ED visits.

**Figure 1:**
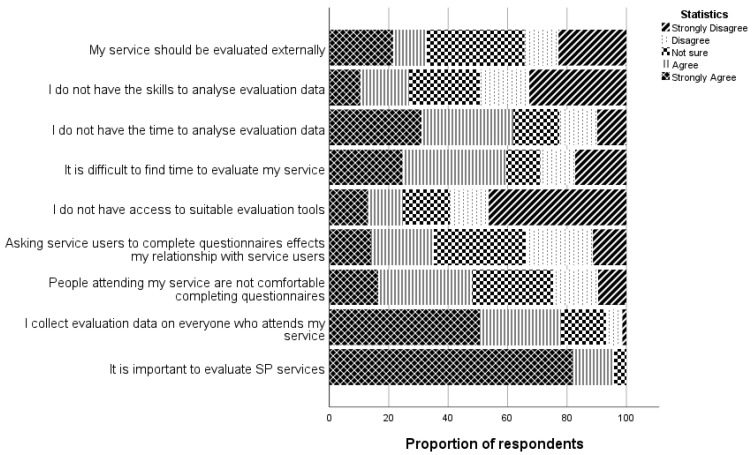
Levels of agreement to barriers and facilitators of evaluation.

Respondents were asked to identify barriers and facilitators to evaluating their social prescribing services on a 10-point Likert scale (1 = Strongly Disagree and 10 = Strongly Agree). Seventy respondents answered this question in full. There was strong agreement on the importance of evaluating SP, with 66/70 (94.3%) respondents either agreeing or strongly agreeing with this statement (Figure 1). In the open-ended question, respondents noted the importance of evaluation: “*We need evaluation for SP to stay and be sustained/funded for people* P60)” and “*Evaluation is a very valuable resource for monitoring and recording impact and improvement of our clients’ health outcomes* (P49)”.

However, over half of respondents (57.1%) also strongly agreed/agreed that collecting evaluation data can be difficult as service users are not always comfortable completing questionnaires. Over one-third of respondents (35.2%)) strongly agreed/agreed that completing evaluation impacts relationship-building with new service users. One participant stated, “*Clients do not want to complete more than one well-being form* (P32)”.

Although 59.5% (*n* = 50/84) strongly disagreed/disagreed that they do not have access to suitable evaluation measures, and in an open question on evaluation, one participant stated, “*I use the GAD-7 but would be interested in hearing about other methods* (P40)”. Respondents also reported having the necessary skills to collect and analyse evaluation data, but 61.9% strongly agreed/agreed to not having sufficient time to analyse data they collect for evaluation purposes. One respondent stated, “*there is a need for admin support to maintain and develop a digital (evaluation) platform. It is a lot to ask LW [link worker] to develop and upkeep a system digitally* (P81)”. When asked if their service should be evaluated by someone external to their organisation, there were differing opinions, with 34.3% (*n* = 24/70) strongly disagreeing/disagreeing with this statement, 32.8% (*n* = 23/70) agreeing/strongly agreeing with the statement and the remaining individuals were unsure.

### 3.3. Community Services and Activities

**Figure 2:**
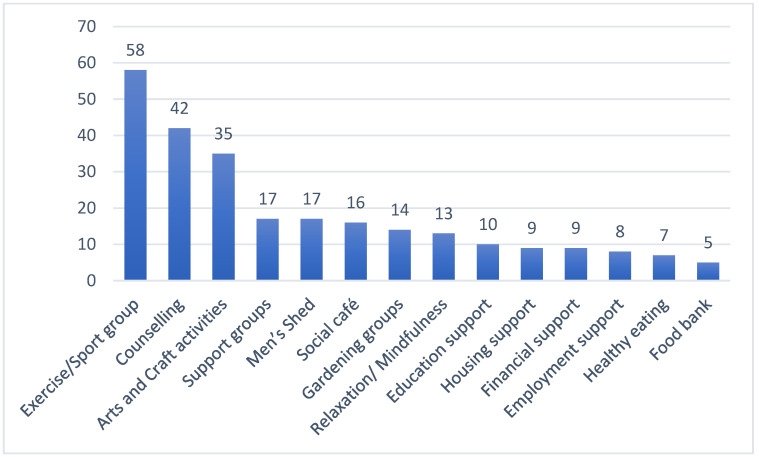
Community services and/or activities to which service users are linked by Social prescribing services.

Respondents were asked to identify community-based activities and services to which they frequently refer their service users. Forty-seven different activities and services were identified by seventy respondents (see Figure 2 for top-ranked activities). The most frequently identified activity was exercise and/or sport-related groups (82.8%, *n* = 58). Counselling was the next most frequently identified community service, followed by arts and crafts activities. Activities and services less frequently used by SP services included referral to healthcare professionals (*n* = 3), volunteering opportunities (*n* = 3) and addiction services (*n* = 2).

### 3.4. Respondents’ Experiences of Evaluation

The final section of the survey was an open-ended question that invited respondents to add any further information/feedback on their experiences of, and recommendations for, evaluation of social prescribing services in Ireland. Twenty-three respondents answered this question. Two categories were identified: recommendations foe evaluation and resources needed for evaluation. These categories and corresponding sub-categories are outlined in Table 6.

Respondents recommended a flexible approach to evaluation as individuals presenting to social prescribing have many different needs. It was recommended that vulnerable service users use conversational methods and service users’ testimonials. Some respondents also identified the importance of allowing sufficient time prior to collecting follow-up evaluations as time is needed for service users to experience the benefit of social prescribing and that this can take many months for some service users. The need for a national approach to evaluation to support consistency across services was identified, and respondents suggested this should be co-designed by all social prescribing stakeholders, particularly service users. Resources needed for evaluation included funding for assessments, suitable systems to collect and analyse evaluation data, and protected time for social prescribing staff to carry out evaluation.

## 4. Discussion

The purpose of this study was to examine the evaluation practices of social prescribing services on the island of Ireland. This study specifically examined what outcomes are currently being evaluated by social prescribing services, how outcomes are measured, and barriers and facilitators to evaluation.

The majority of outcomes being measured in social prescribing services in Ireland are service-user-focused rather than organisational or health-service-focused outcomes. The most frequently measured outcomes are general health and well-being and social connectedness/loneliness. The approaches used most frequently when evaluating these outcomes are questionnaires and general discussion with service users. The most frequently reported questionnaire for measuring both of these two outcomes was the Short Warwick Edinburgh Mental Well-Being (SWEMWBS) Scale [14]. The least frequently measured outcome was satisfaction of those who refer to social prescribing services, with almost 80% of respondents stating they never or rarely measure this service-related outcome. Respondents were in strong agreement on the importance of measuring outcomes of social prescribing in order to support the sustainability of social prescribing services; however, they also stated that evaluation methods need to be flexible and co-designed with all stakeholders and that protected time is needed to complete evaluations. Previous research has also identified the need for protected time for evaluation [9].

### 4.1. Service Profiles

The respondents to this study were either social prescribing link workers/coordinators or individuals involved in managing social prescribing services. Both of these social prescribing roles were included in the study to ensure a comprehensive overview of evaluation practices of social prescribing services. In Ireland, these two positions are involved in all aspects of service delivery and, therefore, are considered well-positioned to provide information on evaluation practices in their services. Most respondents reported that the majority of their referrals were from community mental health professionals, with one of the most commonly reported reasons for referral related to mental health. This is reflected in approaches to evaluation with stress/anxiety and distress identified as one of the most frequently measured outcomes. Counselling was identified as one of the most commonly used community-based services, showing alignment across the profile of individuals referred for social prescribing services, outcomes being measured and the type of activities to which social prescribing staff link service users. This generally aligns with other research on the profile of individuals attending social prescribing services and community-services to which they are linked [16,17].

### 4.2. Approaches to Evaluation

Respondents were asked how often they evaluated outcomes as recommended by national evaluation frameworks (i.e., HSE and NHS). At a minimum, both the NHS and HSE frameworks recommend measuring general health and well-being (GHWB). Almost 90% of respondents reported that they always measure this outcome, and the most frequently identified tool used by respondents to measure this outcome is the Short Warwick Edinburgh Mental Well-Being Scale (SWEMWBS) [14]. General health and well-being was also identified by Sonke et al. as the most commonly measured outcome in their recent mapping review of 87 studies from 13 countries [18]. However, in this current study, the most commonly cited reason for referral to social prescribing was loneliness, and the main approach reported for measuring loneliness was the SWEMWBS. Although loneliness impacts on GHWB, this measure does not specifically measure loneliness. A limitation of having only used a survey to examine social prescribing evaluation approaches in this study is that there is no opportunity to ask respondents to clarify their responses. A future study could include qualitative methods to allow opportunities for elaboration on evaluation methods. 

Sixty-one respondents reported that they always measure service-user participation in social activities. This is not perhaps surprising as loneliness was one of the main reasons for referral identified by respondents, and facilitation of engagement in community-based social activities is a core objective of social prescribing [2]. As with GHWB, the measurement tool used by respondents to assess social participation is the SWEMWBS. However, as none of the questions on the SWEMWBS specifically refer to participation in social activities, it is important to assess if this is the most appropriate questionnaire for measuring this outcome. More research is therefore indicated to identify and test specific measures of social participation for social prescribing services [19].

Although physical health was not identified as one of the top reasons for referral to social prescribing services, it is interesting to note that community-related exercise and sport-related activities and services were the top-ranked activities to which SP staff link service users. There is much research identifying the positive impact of physical activity on mental health, which was one of the top reasons for referrals to respondents’ services. Therefore, referring service users to physical activity classes and programmes reflects evidence-based practice [20]. However, there is also an increasing number of individuals living with chronic health diseases, and increasing physical activity is a core health promotion strategy for the prevention and management of chronic diseases [21]. Therefore, perhaps social prescribing is an under-utilised resource by healthcare professionals with responsibility for individuals living with chronic health diseases. In order to increase physical health-related referrals, social prescribing services could increase healthcare professionals’ awareness of their potential role in facilitating engagement in physical activity. Regarding the assessment of physical health, almost 70% of respondents reported that they always measure physical health. However, only four individuals identified using a specific physical activity measure, the Physical Activity Readiness Questionnaire (PAR-Q), which focuses on readiness to engage in physical activity. Nine individuals stated that they use the SWEMWBS to measure physical health; however, this questionnaire is used to measure general health and well-being and, therefore, is not suited to measuring physical activity. As previously discussed, this finding supports the use of qualitative methods to clarify respondents’ answers to this survey.

The majority of respondents reported collecting data on numerous aspects of the uptake and delivery of their services, including the number of referrals received, the number of individuals who attend their service and who do not attend, and the number and type of contacts between link workers and service users. These are important data to collect to demonstrate to referrers and funders how social prescribing services are being used and by whom. They also inform planning and delivery of future services. However, how these data are used to inform the development of individual services and to whom respondents report these data is unknown. This further supports the use of qualitative methods to gain a deeper understanding of the rationale for the collection of service-related outcomes. One aspect of service delivery not frequently evaluated by respondents is satisfaction of those who refer to social prescribing services. Previous research has identified a reluctance of healthcare professionals to refer their patients to social prescribing services due to a lack of accountability [22]. This would, therefore, indicate the importance of measuring satisfaction levels of those who refer to social prescribing services in order to ensure consistent communication between social prescribing staff and those who refer to social prescribing services, and community-based organisations to whom social prescribing staff refer service users.

Although a large number of respondents reported that they always evaluate person-centred outcomes, including general health and well-being, loneliness, confidence, and physical activity, over half of respondents did not use standardised assessment tools to measure these outcomes, preferring instead to identify service users’ issues through informal discussion. Given the vulnerability and wide range of issues that service users often present with, a core priority for most link workers is to establish a trusting relationship with their service users [23]. In this current study, almost half of the respondents stated that service users were uncomfortable completing evaluation questionnaires and, therefore, a tailored and flexible approach is required based on the profile of their service users. However, there was also feedback from respondents on the importance of developing a national approach to evaluation in order to support consistency in the collection and reporting of evaluation data nationally. These findings demonstrate the complexity of evaluating the impact of social prescribing for all stakeholders, including service users, referrers and staff of community-based organisations to which SP staff link service users. These findings, therefore, support the importance of co-designed national evaluation frameworks informed by all SP stakeholders.

### 4.3. Barriers and Facilitators to Evaluation

Social prescribing staff in this study recognised the importance of evaluation to demonstrate the impact of social prescribing on service users and to support consistent funding to ensure sustainability of their services. There was strong agreement among respondents that they have the skills to collect and analyse data, but they lack time to carry out evaluations and that funders need to recognise this by providing administrative support and user-friendly digital systems. The timing of when to carry out evaluation with service users was also identified as an important factor influencing how and when social prescribing staff carry out evaluation with concerns expressed on how evaluation can impact on relationship-building with clients who are vulnerable and may experience mental health issues and lack confidence. They also highlighted that it takes time to observe changes in the health and well-being of vulnerable individuals with complex health needs. Westlake et al. [24] also stressed the importance of allowing sufficient time between evaluation periods when evaluating scial prescribing services.

Respondents in this study identified the need for flexible approaches to evaluation and that a ‘one-size-fits-all’ approach cannot be used for evaluation given the complexity of people attending SP services and the variability in SP service models on the island of Ireland. In their realist review of evaluation methods, Elliot et al. recommended mixed methods approaches to evaluation [25]. In this current study, there was also a recommendation that a national approach is needed to evaluate guidelines. A national approach would recommend core outcomes to be measured across all social prescribing services. However, flexibility is also needed to enable services to incorporate tailored aspects of evaluation to align with the model and focus of their particular service. One measure that could possibly provide uniformity in data collected nationally but still facilitate differences across services is Measure Yourself Concerns and Well-Being (MYCaW) [26]. This person-centred measure allows a service user to identify the two most important issues impacting on their health and well-being at the time of referral. The MYCaW enables people to identify what matters to them rather than being restricted to a pre-determined list of items to choose from, as is often the norm in standardised health-related outcome measures. Although the MYCaW is widely used in social prescribing services in other countries [25], in this current study, only three respondents identified using this measure. This indicates the need to test the useability and acceptability of MYCaW across different services on the island of Ireland.

## 5. Strengths and Limitations

This is the first survey to explore the evaluation practices of social prescribing services on the island of Ireland. Eighty-six surveys were completed, eighty-four of which were included in the final analysis. Based on the number of services listed on the directory of services on the All-Ireland social prescribing Network (AISPN) website, at the time of the study, it appears that the majority of services are represented in this study. Due to the similarities between the profile of services reported in this survey and international literature on social prescribing services, it is expected that the findings from this study may be transferable and informative to social prescribing services in other countries with similar service delivery models as those described in this study.

However, there are some limitations to the study. Respondents of the survey were not asked to identify their service for reasons of data protection, and that information on evaluation practices could be considered sensitive by some services. Therefore, it was not possible for the study team to ensure that all services listed on the AISPN website were included in the study or that only one survey was completed by individual services, leading to a risk of duplication. Attempts were made by the study team to mitigate against more than one survey per service being included in the study. These included clearly stating this in the participant information leaflet, making regular announcements during the two-day AISPN conference to reiterate this instruction. Additionally, there was a chance that social prescribing staff who completed the survey during their attendance at the AISPN conference may also have completed the online survey, leading to duplication. To prevent this, instructions were clearly included at the beginning of the online survey, requesting individuals not to complete the online survey if they had already completed a hard copy of the survey at the AISPN conference.

## 6. Conclusions

Given the absence of robust evaluation of social prescribing, the purpose of this study was to explore evaluation practices in social prescribing services on the island of Ireland. As with other research examining evaluation practices of social prescribing services, considerable variation was noted in both the outcomes measured and how they are measured. Considering the variability in who attends social prescribing services and where and how services are delivered, there is a need for flexibility in methods used for evaluating outcomes of social prescribing. However, consistency in evaluation across services would help to establish the impact of social prescribing and identify preferred models for the delivery of social prescribing services. This indicates the need to develop a core outcome set for social prescribing.

## Figures and Tables

**Table 1:** Profile of Social Prescribing Services.

		*n* (%)
**Location**	NI ^1^	42 (50%)
	ROI ^2^	42 (50%)
**Role in SP service**	SP Link Worker ^3^	40 (47.1%)
	SP Coordinator ^4^	22 (25.9%)
	Other	22 (27.0%)
**Location of SP service**	Community Health Centre	27 (34.1%)
	Family Resource Centre	25 (31.6%)
	Partnerships organisations	12 (15.1%)
	Other	26 (33%)
**Source of referrals to SP services**	Com MH Service ^5^	69 (82.1%)
	GP ^6^	67 (79.8%)
	Social Worker	59 (69.4%)
	Self-Referral	57 (67.1%)
	Other	32 (38.1%)
**Age (years) of individuals referred to SP**	Less than 18	12 (14.1%)
	19–30	74 (87.1%)
	31–65	81 (95.3%)
	66+	80 (94.1%)
**Reasons for referral**	Mental Health	117
	Social Health	104
	Physical Health	59
	Other	341

^1^ Northern Ireland, ^2^ Republic of Ireland, ^3^ Social Prescribing Link Worker, ^4^ Social Prescribing Coordinator, ^5^ Community Mental Health Service, ^6^ General Practitioner.

**Table 2:** Commonly cited reasons for referral to SP services.

Mental Health	Social Health	Physical Health	Other
Unspecified mental health (*n* = 32) Anxiety (*n* = 29) Depression (*n* = 28) Bereavement (*n* = 9)	Loneliness (*n* = 80) Befriending (*n* = 17) Family Support (*n* = 5) Relationship breakdown *(n* = 3)	Chronic disease management (*n* = 19) Pain management (*n* = 16) Exercise (*n* = 14) Nutritional Support (*n* = 4) Falls prevention (*n* = 2)	Link to new activities (unspecified) (*n* = 8) Housing support (*n* = 5) Education/employment/career advice (*n* = 3) Financial advice (*n* = 2)

**Table 3:** Approaches to Evaluation: Person-centred Outcomes *.

	How Often		Approaches Used *n* (%)	Frequently Used Questionnaires *n* (%)
	Never/Occasionally *n* (%)	Always *n* (%)		
**General Health and Well-being**	8/74 (10.8%)	66/74 (89.2%)	Questionnaire *n* = 42/60 (70%)	SWEMWBS ^1^ *n* = 28/50 (56%)
			Informal discussion *n* = 11/60 (18.3%)	Outcome Star *n* = 13/50 (26%)
**Social Connectedness/Loneliness**	12/75 (16%)	63/75 (84%)	Questionnaire *n* = 31/56 (55.4%)	SWEMWBS *n* = 26/42 (61.9%)
			Informal discussion *n* = 19/56 (33.9%)	Outcome Star *n* = 13/42 (31.0%)
**Participation in Social Activities**	15/76 (19.7%)	61/76 (80.3%)	Questionnaire *n* = 21/47 (44.7%)	SWEMWBS *n* = 11/36 (30.6%)
			Informal discussion *n* = 17/47 (36.2%)	Outcome star *n* = 11/36 (30.6%)
**Stress/Anxiety/Distress**	19/75 (25.3%)	56/75 (74.7%)	Questionnaire *n* = 37/53 (43.0%)	SWEMWBS *n* = 30/45 (66.6%)
			Informal discussion n= 11/53 (20.7%)	Outcome star *n* = 10/45 (22.2%)
**Confidence/Self-Esteem**	20/75 (26.6%)	55/75 (73.4%)	Questionnaire *n* = 40/59 (67.8%)	SWEMWBS *n* = 29/47 (61.7%)
			Informal discussion *n* = 14/59 (23.7%)	Outcome star *n* = 12/47 (25.5%)
**Physical Activity levels/Physical activity Health**	23/75 (30.6%)	52/75 (69.4%)	Informal Discussion *n* = 20/45 (44.4%)	SWEMWBS *n* = 9/30 (30.0%)
			Questionnaire *n* = 20/45 (44.4%)	Outcome Star *n* = 8/30 (26.7%)
				PAR-Q ^2^ *n* = 4/30 (13.3%)
**Depression**	25/76 (32.9%)	51/76 (67.1%)	Questionnaire *n* = 28/48 (58.3%)	SWEMWBS *n* = 25/36 (69.5%)
			Informal discussion *n* = 15/48 (31.3%)	Outcome Star *n* = 11/36 (30.6%)
**Change in financial situation**	37/75 (49.3%)	38/75 (50.7%)	Informal Discussion *n* = 16/35 (45.7%)	SWEMWBS *n* = 9/23 (39.1%)
			Questionnaire *n* = 11/35 (31.4%)	Outcome star *n* = 11/23 (47.7%)
**Change in Employment Status**	37/74 (50%)	37/74 (50.0%)	Questionnaire *n* = 14/35 (40%)	Outcome star *n* = 11/24 (45.8%)
			Informal Discussion *n* =13/35 (37.1%)	SWEMWBS *n* = 9/24 (37.5%)

* There was significant variation in the number of respondents to each question; ^1^ Short Warwick–Edinburgh Mental Well-being Scale; ^2^ Physical Activity Readiness Questionnaire.

**Table 4:** Standardised questionnaires used by respondents in their services.

Questionnaire	Standardised Measures Used in SP Services *n* = 84
Short Warwick–Edinburgh Mental Well-being Scale (SWEMWEBS)	38 (45.2%)
Outcome Star	18 (21.4%)
Pillars of Positive Health (POPH)	9 (10.7%)
World Health Organisation Well-Being Index (WHO-5)	8 (9.5%)
The Wheel of Life	4 (4.7%)
Physical Activity Readiness Questionnaire (PAR-Q)	4 (4.7%)
Measure Yourself Concerns and Well-being (MYCaW)	3 (3.5%)
General Anxiety Disorder-7 (GAD-7)	2 (2.3%)
Euro QoL 5D *	2 (2.3%)
UCLA Loneliness Scale	1 (1.2%)

* Not specified whether Euro QoL 5D-5L or Euro QoL 5D-3L is used.

**Table 5:** Approaches to Evaluation: SP Service-Related Outcomes *.

Outcomes Related to Service Uptake and Delivery	How Often		Approaches Used to Measure Service-Related Outcomes *n* (%)
	Never/Occasionally *n* (%)	Always *n* (%)	
**Number of referrals you received for your service**	13/73 (17.8%)	59/73 (80.8%)	Digital platform 20/31 (64.5%)
**Number of individuals referred to your service, but do not attend**	19/73 (26.0%)	54/73 (74.0%)	Digital 19/28 (67.9%)
**Record of activities and/or support services accessed by individuals following meetings with LW ^1^**	15/73 (20.5%)	58/73 (79.5%)	Digital 18/34 (52.9%)
**Type of contact between LW and individuals attending your SP ^2^ service**	18/76 (23.7%)	58/76 (76.3%)	Digital 19/37 (51.4%)
**Number of contacts between LW and individuals attending your SP service**	21/75 (28.0%)	54/75 (72.0%)	Digital 17/34 (50%)
**Satisfaction of individuals who attend your service**	36/74 (48.6%)	38/74 (51.4%)	Questionnaire 11/28 (39.3%)
**Case studies of individuals who attend your service**	46/73 (67.1%)	27/73 (36.9%)	Digital 9/19 (47.4%)
**Satisfaction of HCP and other organisations who refer individuals to your service**	58/73 (79.5%)	15/73 (20.5%)	Digital 5/14 (35.7%)

* There was significant variation in the number of respondents, and response rates, for each question; ^1^ Link Worker; ^2^ Social Prescribing.

**Table 6:** Content analysis of open-ended questions on recommendations for evaluation.

Category	Sub-Categories	Participant Quotes
**Recommendations for evaluation**	National approach	P24 *Evaluation tools should be decided nationally so that data can be shared and understood* P47 *There needs to be consistency in relation to the collection of data and how it is reported on nationally*
	Flexible approaches	P11 E*valuation systems need to be widespread and not a ‘one-size fits all’ as there are as many individual ways of recording evaluations as there are individuals* P25 *It need to be less generic, direct evaluation tools for different age groups. One size does not fit all*
	Qualitative evaluation methods	P49 *more qual data needs to be captured along with quant data* P61 *we need qual tools that are suitable for people with low or no literacy where trauma or panic impacts on ability to think and process—we need to allow people to tell their story safely and confidentially which is accepting/appropriate to funders*
	Timing of evaluation	P81 *I find using evaluation tools easier when the client first engages. Social prescribing is not a service with a set timeframe so a relationship can be ongoing and carrying out a ‘post-assessment’ is challenging. Often once a client is engaging and happy, the priority is to support new clients.* P81 *The relationship may have changed over time, and you can be working with a client more over the phone so it’s less formal and therefore more difficult to implement an exit interview. Also, there may be ongoing challenges meaning the relationship continues in a different vein or with a different focus.*
	Multiple perspectives	P32 *It would be great to record the LWs assessment of the person’s improvement also. Important to record—is the person now more engaged in their communities and do they plan to continue?* P86 *Feedback from the services/supports/activities referred to will be useful in guiding future referrals*.
	Co-design evaluation methods	P78 *I do feel there is a system of evaluation which should be developed in conjunction with social prescribers*
**Resources needed**	Measurement tools	P47 *there needs to be wide access to evaluation tools.* P79 *They have no formal evaluation measures available to me and no digital system that they will provide.*
	Funding	P61 *need to do invest in quality evidence methods*
	Time to evaluate change in service users	P69 *The depth of work is not being measured—some people just need information some people take months to build up trust/This is not being measured.* P69 *The impact is much more difficult to measure as the outcomes may be long term and may not be obvious or measurable in the short term. This I think needs further thought, and to ask social prescribers to measure real impact in 8 weeks is not achievable and will not give accurate results.*
	Time for LW to evaluate	P79 *Time constraints stops me from being able to evaluate case studies of individuals who attend my service. I work 11 h weekly with a heavy case load and large waiting lists, high number of referrals, and no opportunity to assess needs and evaluate the service properly.* P38 *Evaluating is very time consuming so this needs to be considered*

## Data Availability

The data presented in this study are available on reasonable request from the corresponding author.
